# Genetically modified crops do not present variations in pollen viability and morphology when compared to their conventional counterparts

**DOI:** 10.1371/journal.pone.0285079

**Published:** 2023-05-01

**Authors:** Daniel Soares, Hallison Vertuan, Fabiana Bacalhau, Marcia José, Augusto Crivellari, Gustavo G. Belchior, Geraldo U. Berger

**Affiliations:** Regulatory Science, Bayer Crop Science, São Paulo, SP, Brazil; Jeju National University, REPUBLIC OF KOREA

## Abstract

Modern agricultural biotechnologies, such as those derived from genetic modification, are solutions that can enable an increase in food production, lead to more efficient use of natural resources, and promote environmental impact reduction. Crops with altered genetic materials have been extensively subjected to safety assessments to fulfill regulatory requirements prior to commercialization. The Brazilian National Technical Biosafety Commission (CTNBio) provides provisions for commercial release of transgenic crops in Brazil, including requiring information on pollen dispersion ability as part of environmental risk assessment, which includes pollen viability and morphology studies. Here we present the pollen viability and morphology of non-transgenic conventional materials, single-event genetically modified (GM) products, and stacked GM products from soybean, maize and cotton cultivated in Brazil. Microscopical observation of stained pollen grain was conducted to determine the percentage of pollen viability as well as pollen morphology, which is assessed by measuring pollen grain diameter. The pollen viability and diameter of GM soybean, maize and cotton, evaluated across a number of GM events in each crop, were similar to the conventional non-GM counterparts. Pollen characterization data contributed to the detailed phenotypic description of GM crops, supporting the conclusion that the studied events were not fundamentally different from the conventional control.

## Introduction

Plants derived from modern biotechnology have been one of the fastest adopted crop technologies in history, with approximately 70 countries utilizing biotechnology-derived crops for cultivation and importation for food, feed, and processing. Genetically modified (GM) crops offer sustainable solutions to agriculture, especially important with increasing challenges brought by climate change and a growing population [1]. Conventional plant breeding has successfully improved crops through selection practices that capture genetic gains, even though information on the specific genes and genetic networks that contribute to the desired agronomic characteristics has historically been limited. GM crops providing agronomic traits, such as herbicide tolerance (HT) and insect resistance (IR), have shown significant commercial relevance due to yield protection, environmental benefits through reduced pesticide use, greater flexibility in crop management practices, and increased crop performance, allowing farmers to overcome challenges and meet their needs [2, 3].

Biotechnology-derived crops have been adopted for more than 25 years, and their extensive biosafety assessment has consistently concluded that they do not pose an increased food/feed or environmental risk when compared to their conventional counterparts [4]. Additionally, commercially available GM crops have been determined to pose negligible ecological risk via invasion of non-agricultural habitats based on the absence of phenotypic differences with non-transgenic comparators that would indicate an increase in invasiveness potential [5, 6]. Environmental Risk Assessment (ERA) field trials are designed to test whether the invasiveness potential of the transgenic crop has increased due to unintended effects of transformation and introgression of the biotechnology-derived trait [7].

Evidence in previous environmental risk assessment studies have shown that both single-event GM and stacked-event GM products do not represent risks for human, animal, and environment. [8].Trials performed across Brazil demonstrated there were no differences in agronomic and phenotypic plant characteristics for both single-event and stacked products for soybean, maize and cotton when compared to their conventional counterparts [9]. Moreover, there is scientific consensus that genetic engineering methods are safe [10–19]. Even though years of research and decades of commercialization of GM crops show that these products are safe and raise no additional biosafety concerns, risk assessments for these products are still required in many contexts, including ERA.

The Brazilian National Technical Biosafety Commission (CTNBio) is the regulatory agency for technical and advisory matters pertaining to GM crops in Brazil. Applicants seeking to commercialize biotechnology-derived crops should provide CTNBio with molecular characterization, product biosafety data to human and animal health, and environmental data from an ERA perspective.

As part of the ERA to evaluate the biosafety of GM crops, the Brazilian regulatory agency requests pollen evaluation of GM plants compared to their conventional counterparts to be presented as part of the risk assessment evaluation for biotechnology-derived crops. Here, we present pollen viability and morphology (diameter) data collected from non-GM conventional, single-event GM and stacked-event GM products from soybean (*Glycine max*), maize (*Zea mays*) and cotton (*Gossypium hirsutum*) cultivated in different, representative agricultural regions in Brazil.

## Materials and methods

Field trials were conducted in five different seasons (from 2013 to 2017) in Brazil at Bayer Experimental Stations in Santa Cruz das Palmeiras, São Paulo (SP) State and Rolândia, Paraná (PR) State for field pollen collection and analysis. All trials were conducted in a randomized block design with four replications. Crop materials included GM soybean (*Glycine max* L.), GM maize (*Zea mays* L.) and GM cotton (*Gossypium hirsutum* L.) products, described in Table 1. Along with each crop and event combination, a conventional control (with the same genetic background as the GM event) and three to four commercial references available in the market were cultivated. The materials (GM, conventional control and references), cultivation season and location are presented in S1 Table.

**Table 1:** Single and stacked events assessed for pollen viability and morphology.

**Crop**	**Single/stacked products**	**Trait**	**Transgenic gene product(s)**
Soybean	MON 87751	Insect resistance	IR: Cry1A.105, Cry2Ab2
	MON 87708	Herbicide tolerance	HT: DMO
	MON 87701	Insect resistance	IR: Cry1Ac
	MON 89788	Herbicide tolerance	HT: CP4 EPSPS
	MON 87708 × MON 89788	Herbicide tolerance	HT: DMO, CP4 EPSPS
	MON 87751 × MON 87701 × MON 87708 × MON 89788 (Soybean stack)	Insect resistance and herbicide tolerance	IR: Cry1A.105, Cry2Ab2, Cry1Ac HT: DMO, CP4 EPSPS
Maize	MON 87411	Insect resistance and herbicide tolerance	IR: Cry3Bb1, *DvSnf7* HT: CP4 EPSPS
	MON 87427	Herbicide tolerance	HT: CP4 EPSPS
	MON 89034	Insect resistance	IR: Cry1A.105, Cry2Ab2
	MON 89034 × MIR162	Insect resistance	IR: Cry1A.105, Cry2Ab2, Vip3Aa
	MON 87427 × MON 89034 × MIR162 × MON 87411 (Maize stack)	Insect resistance and herbicide tolerance	IR: Cry1A.105, Cry2Ab2, Vip3Aa, Cry3Bb1, *DvSnf7* HT: CP4 EPSPS
	MON 87429	Herbicide tolerance	HT : CP4 EPSPS, DMO, FT_T, PAT
	MON 95379	Insect resistance	IR: Cry1B.868, Cry1Da_7
Cotton	MON 88701	Herbicide tolerance	HT: DMO, PAT
	MON 88913 × MON 88701	Herbicide tolerance	HT: DMO, CP4 EPSPS, PAT
	COT102 × MON 15985 × MON 88913 × MON 88701 (Cotton stack)	Insect resistance and herbicide tolerance	HT: CP4 EPSPS, DMO, PAT IR: Vip3Aa, Cry1Ac, Cry2Ab2

Products are indicated by their event codes. Each biotechnology-derived trait (IR: insect resistance; HT: herbicide tolerance) is indicated per single or stacked product, as well as corresponding transgenic gene product. CP4 EPSPS: *Agrobacterium tumefaciens* (strain CP4) 5-enolpyruvylshikimate-3-phosphate synthase (tolerance to glyphosate herbicide); Cry (various proteins): *Bacillus thuringiensis* (different strains), Cry δ-endotoxins (resistance to lepidopteran/coleopteran insects); DMO: *Stenotrophomonas maltophilia* (strain DI-6), dicamba mono-oxygenase (tolerance to dicamba herbicide); *DVSnf7*: *Diabrotica virgifera virgifera* double-stranded RNA transcript containing a 240 bp fragment of the *Diabrotica* species *Snf7* gene (resistance to specific Coleopteran insects); PAT: *Streptomyces hygrosopicus* phosphinothricin N-acetyltransferase (tolerance to glufosinate herbicide); Vip3Aa: *Bacillus thuringiensis* (strain AB88) vegetative insecticidal protein (lepidopteran insect resistance); FT_T: *Sphingobium herbicidovorans* dioxygenase protein (tolerance to 2,4-D and FOPs herbicides). All gene products are proteins, except for the double-stranded RNA molecule *DvSnf7*.

After event transformation and selection in soybean, maize and cotton, conventional breeding techniques were used to obtain the introgressed variety (soybean and cotton) or hybrid (maize). Backcrossing was utilized to obtain the event in the desired variety or inbred background prior to field testing. Generally, the donor parent contains the gene of interest that can be introgressed via backcrossing into a variety or inbred. Backcross breeding allows the transfer of one or more genes of interest from a donor parent into the background of an improved variety/inbred to recover the recurrent parent genome by eliminating the undesirable genes of the transformation line. The backcross populations are made by crossing the recurrent parent with the donor parent to produce the F1 generation, crossing the F1 generation again with the recurrent parent (BC1F1) [20] and then conducting successive backcrosses to recover the recurrent parent genome while retaining the desired characteristic (*e*.*g*., insect resistance or herbicide tolerance). To obtain the stack products, classical breeding crosses and selection were made, where the transgenes of interest came from paternal, maternal or both parents, depending on the stack product. Specifically for maize, after inbred introgression, the single cross hybrid (F1) was produced for planting in the trials. In the cultivation of GM plants in the field, both the event and its respective conventional control were properly evaluated.

The events and corresponding conventional controls in these trials were introgressed and cultivated in a 5.9 maturity group soybean variety, a 130 relative maturity maize hybrid, and a mid-maturity cotton variety. These selected genetic backgrounds were appropriate for the geographic regions in which the trials were conducted. Commercial reference materials for each trial were selected to represent a range of maturities and genetic backgrounds available on the market and to be suitable to the geographic regions where they were cultivated.

### Sample collection

Pollen grains from GM, conventional counterpart and commercial reference plants were collected randomly in each plot. Plots of each material were replicated four times in a randomized complete block design.

For soybean sampling, flowers were collected at the R2 growth stage. Newly opened individual flowers were selected for pollen collection. Soybean flowers were kept cool with ice packs upon collection to avoid decrease in viability. One sample of approximately 20 flowers was collected per plot. Fresh flowers were transferred to the laboratory where pollen was extracted from anthers using forceps and stained prior to microscopic observation.

For maize plants, pollen was collected between the VT and R1 growth stages. Pollen samples were collected from three different tassels per plot and fixed in the stain solution as soon as possible after collection, as viability decreases rapidly when removed from the plant. Three subsamples were collected per plot, each subsample consisting of pollen from the tassel of a single plant. In the field, pollen samples were sieved through a wire mesh screen (#40 mesh sieve) to remove excess debris and anthers and were stained within 30 minutes of collection.

Cotton plants were sampled at approximately the peak bloom growth stage. Newly opened white cotton flowers were selected to ensure fresh pollen was collected. Three subsamples were collected per plot, each subsample consisting of a single flower from a randomly selected plant. Pollen grains were collected from each subsample and fixed in the stain solution immediately after collection.

### Staining procedure and laboratory analyses

The pollen viability and morphology analyses were carried out in the laboratory in Santa Cruz das Palmeiras, SP, Brazil and Rolândia, PR, Brazil. The evaluation was performed using a staining protocol [21]. For soybean, viable pollen grains were stained dark blue and light blue for non-viable grains. For maize, viable pollen grains were stained purple and greenish for non-viable grains. For cotton, viable pollen grains were stained dark blue and greenish for non-viable grains. After solution preparation and pollen collection, pollen grains were placed in the staining solution, diluted to 1:5 (1 part stain:5 parts distilled water) for maize and cotton and undiluted for soybean, and mixed thoroughly.

**Fig 1:**
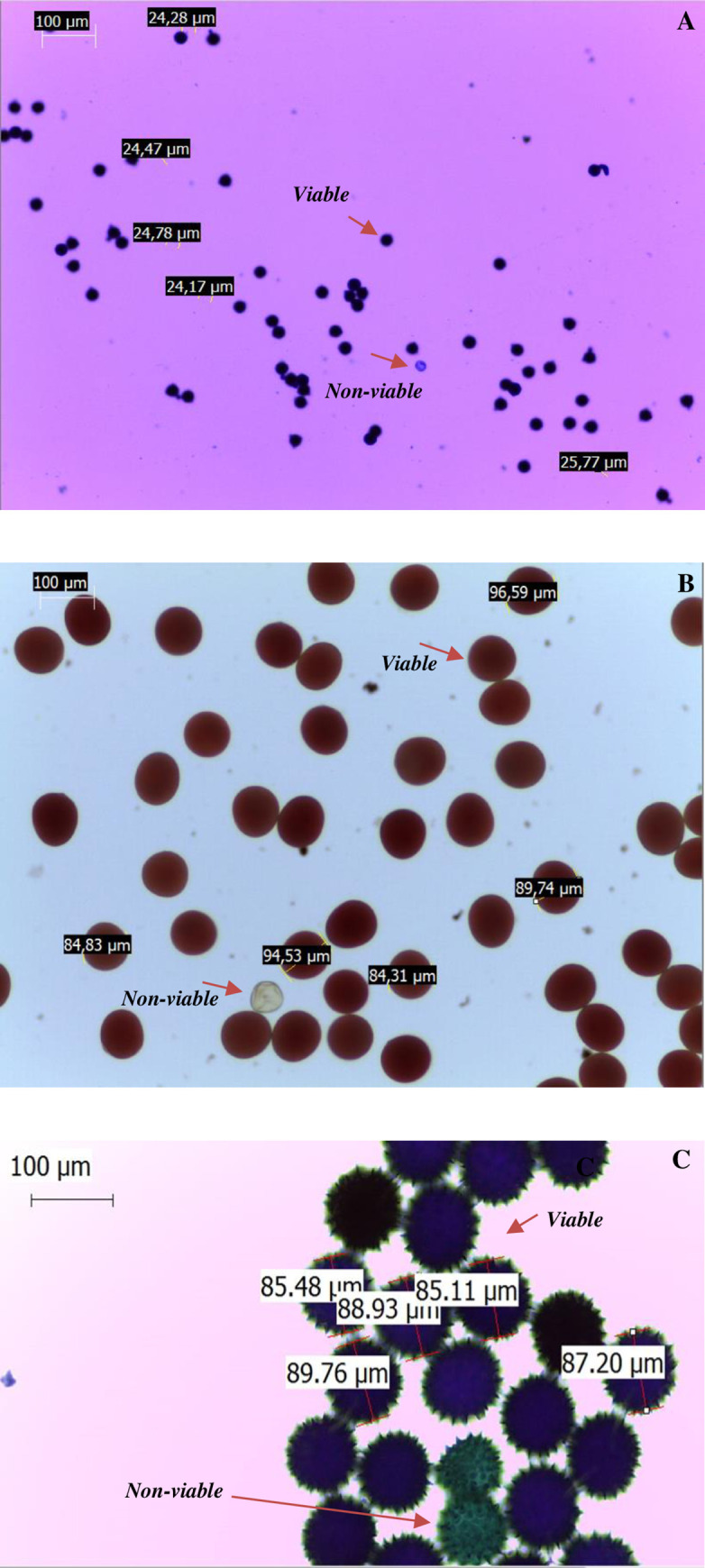
Optical microscope observation for analysis of pollen viability for soybean (A), maize (B), and cotton (C) at 100x magnification.

Staining solution-to-pollen volume ratio (v/v) was of at least 2:1 for maize and cotton. Soybean and maize pollen were stained for at least 10 minutes before microscope observation; cotton pollen was stained for at least 20 hours at room temperature due to pollen wall thickness. Approximately 20–30 μL of each stained pollen subsample was added to a glass slide for observation under the microscope. Fig 1 represents pollen samples from soybean, maize, and cotton, respectively, analyzed under an optical microscope.

### Pollen viability and diameter evaluation

The number of viable and non-viable pollen grains was determined by visual counting, from a single field or multiple fields of view, with a microscope field of view (FOV) showing the pollen grains at a total magnification of 100x (10x objective lens magnification × 10x eyepiece lens magnification). Approximately 75–100 pollen grains were counted for viability per subsample. All pollen grains within the FOV were counted, even if the number of pollen grains exceeded 100. Viable and non-viable pollen grains were counted, and the percentage of viable pollen grains was estimated.

Microscopic Leica IC80 HD images were used for pollen diameter measurements. Microscopic images were taken of at least 1 subsample per replicate. Using the saved pollen sample image(s) from 1 randomly selected subsample from each plot, the diameter of 20 viable pollen grains were determined.

### Variance components analyses

Analysis of variance (ANOVA) was performed and pairwise differences between the GM events and their conventional counterparts were tested by *t-test* at the 5% level of significance (α = 0.05). The analysis was conducted according to the following model for a randomized complete block design, using JMP^®^ 12 software:

$$Y_{ij}=\mu +R_{i}+M_{j}+\varepsilon_{ij}$$

where:

Y_ij_ is the observed response for the j^th^ material (GM and non-GM) in the i^th^ replicate;

μ is the overall mean;

R_i_ is the random effect of the i^th^ replicate;

M_j_ is the fixed effect of the j^th^ material (GM and non-GM);

ε_ij_ is the residual error.

Reference ranges were determined from the minimum and maximum mean values observed for commercial references materials. When significant differences between the GM event and conventional control were detected, the GM mean value was compared to the reference range and determined if the value was within the range. The commercial references cultivated in these studies represented the natural variability for pollen viability and morphology (diameter) in each crop.

ERA studies on GM crops typically make use of pairwise comparisons between the GM product and its conventional counterpart to evaluate mean values. This statistical analysis approach used to compare the means between GM crops versus conventional control has been demonstrated in previous publications [8, 9, 22].

## Results and discussion

**Fig 2:**
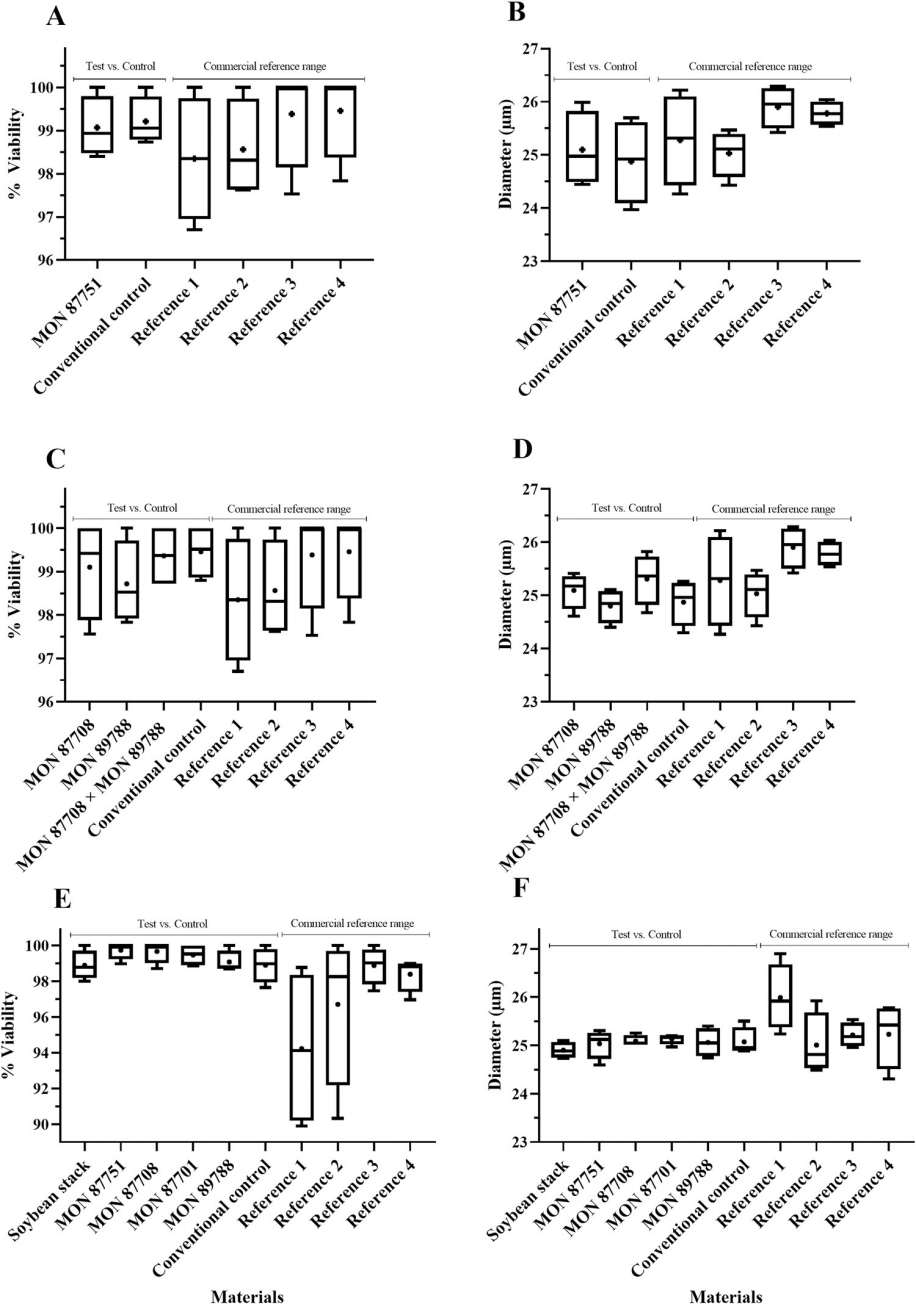
Soybean pollen viability (%) (A, C, E) and diameter (μm) (B, D, F) evaluation in single and stacked event products. Test materials (single and stacked event products) were compared to conventional controls (conventional counterparts). Commercial references provided the minimum and maximum mean values used to create a crop-specific range. Data are presented as box and whisker plots, where the plus symbol (+) represents the mean value, the upper limit is the maximum value, and the lower limit is the minimum value for a given material. Commercial reference material names, trial locations and years can be found in S1 Table. “Soybean stack” refers to the stacked event MON 87751 × MON 87701 × MON 87708 × MON 89788. No significant differences (α = 0.05) were detected between any test and conventional control comparisons.

**Fig 3:**
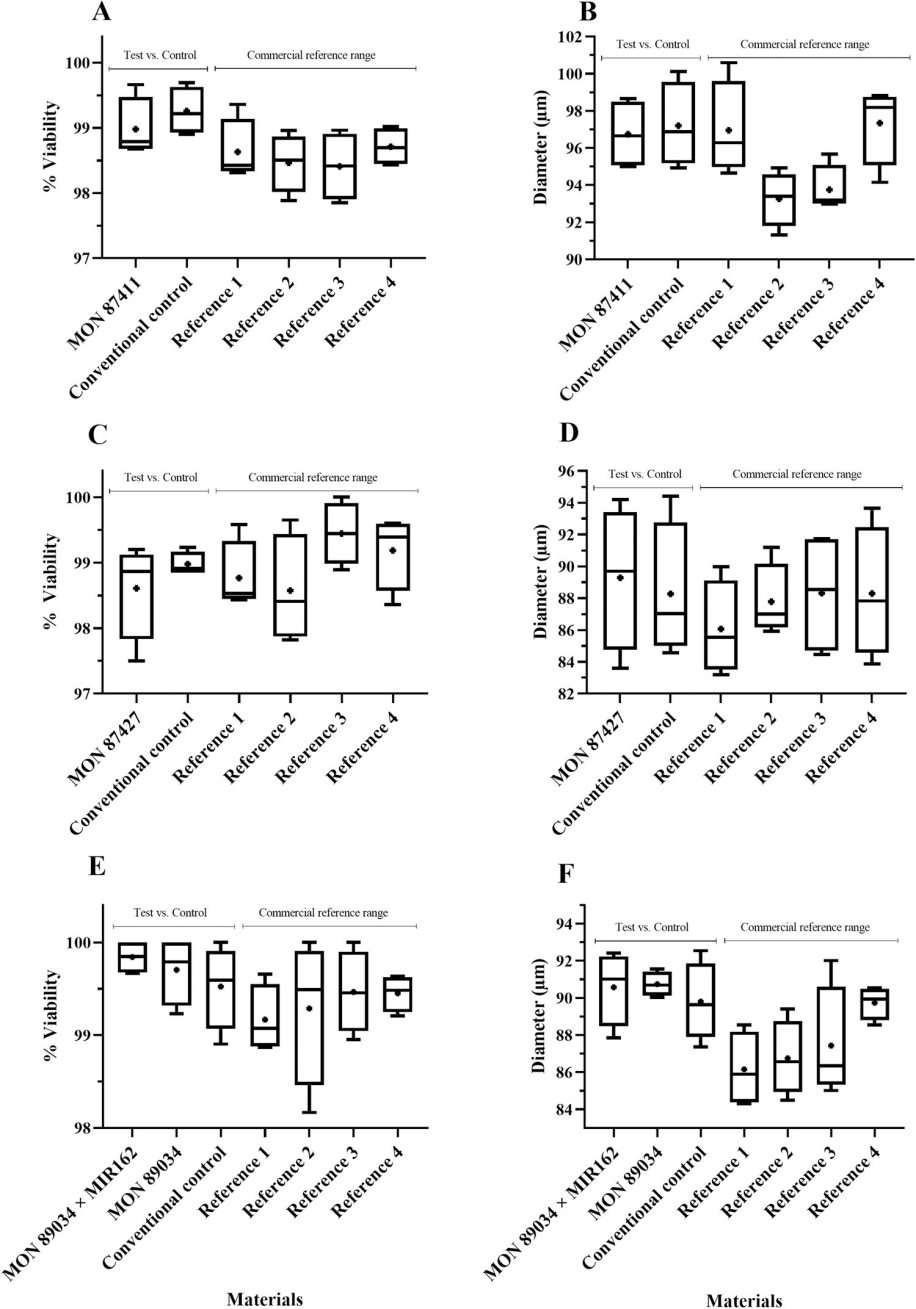
Maize pollen viability (%) (A, C, E) and diameter (μm) (B, D, F) evaluation in single and stacked event products. Test materials (single and stacked event products) were compared to conventional controls (conventional counterparts). Commercial references provided the minimum and maximum mean values used to create a crop-specific range. Data are presented as box and whisker plots, where the plus symbol (+) represents the mean value, the upper limit is the maximum value, and the lower limit is the minimum value for a given material. Commercial reference material names, trial locations and years can be found in S1 Table. No significant differences (α = 0.05) were detected between any test and conventional control comparisons.

**Fig 4:**
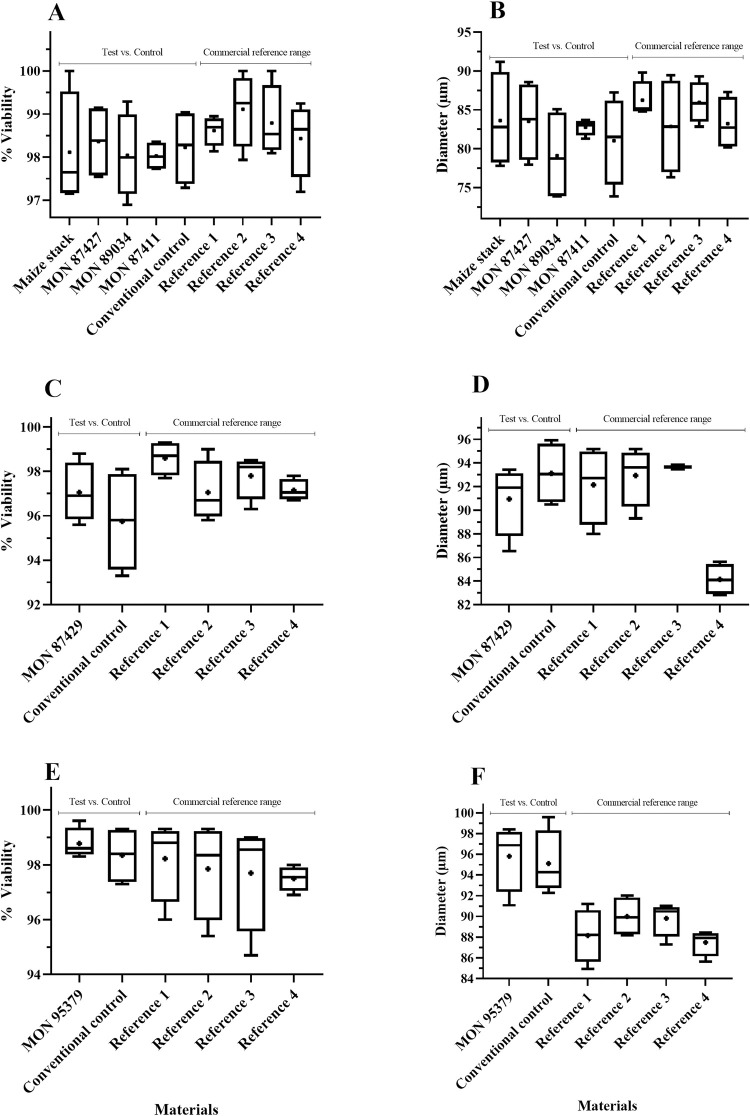
Maize pollen viability (%) (A, C, E) and diameter (μm) (B, D, F) evaluation in single and stacked event products. Test materials (single and stacked event products) were compared to conventional controls (conventional counterparts). Commercial references provided the minimum and maximum mean values used to create a crop-specific range. Data are presented as box and whisker plots, where the plus symbol (+) represents the mean value, the upper limit is the maximum value, and the lower limit is the minimum value for a given material. Commercial reference material names, trial locations and years can be found in S1 Table. “Maize stack” refers to the stacked event MON 87427 × MON 89034 × MIR162 × MON 87411. No significant differences (α = 0.05) were detected between any test and conventional control comparisons.

**Fig 5:**
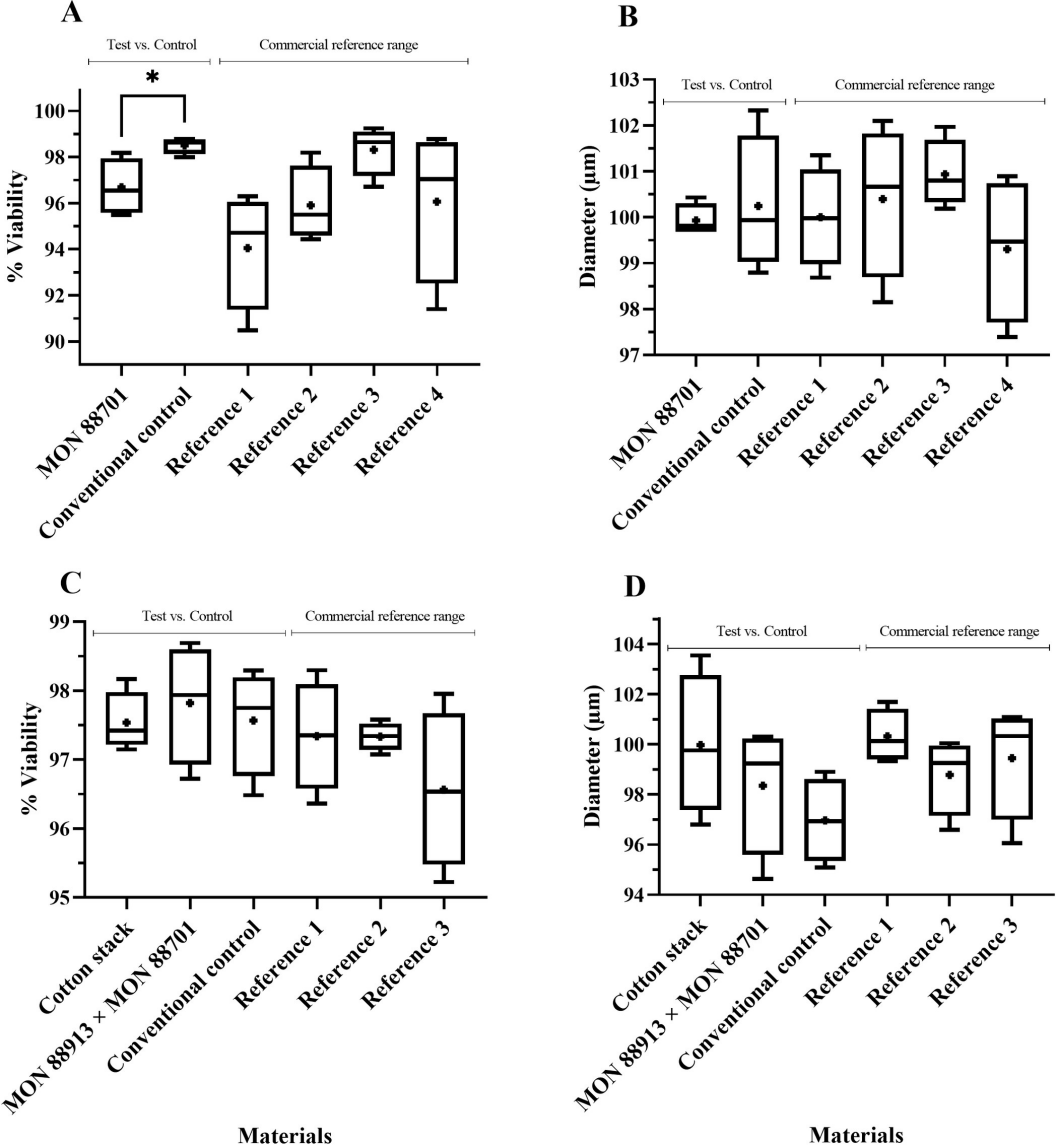
Cotton pollen viability (%) (A, C) and diameter (μm) (B, D) evaluation in single and stacked event products. Test materials (single and stacked event products) were compared to conventional controls (conventional counterparts). Commercial references provided the minimum and maximum mean values used to create a crop-specific range. Data are presented as box and whisker plots, where the plus symbol (+) represents the mean value, the upper limit is the maximum value, and the lower limit is the minimum value for a given material. Commercial reference material names, trial locations and years can be found in S1 Table. “Cotton stack” refers to the stacked event COT102 × MON 15985 × MON 88913 × MON 88701. *Indicates significant difference (p≤0.05) between GM and conventional control.

For soybean, maize and cotton crops, the results were expressed as averages of the percentage of pollen viability and the diameter of pollen grains (morphology) in an evaluation between GM crops (single events and stacked products) and their respective conventional counterparts. Commercial references were included to establish a natural range of pollen viability and morphology of the crop within the marketplace. The results are presented in Figs 2–5.

There were no significant differences in pollen viability (%) and morphology (diameter) across the three GM crops tested compared to their conventional controls, except for the viability percentage of MON 88701 cotton, which presented a lower mean value when compared to the conventional control (96.85 vs. 98.55%). However, the MON 88701 mean value was within the range of commercial reference means tested in the same trial (Fig 5A). It is important to note that commercial references (varieties/hybrids) are included in ERA studies to observe the variability that already occurs for each agronomic characteristic in each particular crop [5, 23, 24].

Agronomic endpoints related to reproduction (pollen viability, pollination rates, etc.) can be used to inform whether a GM trait has increased the potential for outcrossing, and provides information on the crop biology [25] and the potential to increase weediness or invasiveness due to reproduction rates [26].

Using a similar methodology and working with different soybean events, a previous study also observed no significant differences in pollen characteristics between GM soybean and their respective non-GM conventional controls [27]. An ERA study performed in Brazil assessed agronomic characteristics, including pollen viability, for a single-event and a stacked maize product. Pollen viability, as well the agronomic characteristics for all GM maize materials were statistically indistinguishable from the non-GM used as a conventional control [13]. A study with a herbicide tolerant GM cotton demonstrated that no significant differences were detected between the HT event compared to the conventional control when the floral morphology and pollen viability were assessed [28]. These results corroborate the results found in this paper, demonstrating that single events and stacked products across multiple crops do not change pollen viability and diameter when compared to a conventional control, and do not pose relevant pathways to impact traits related to reproduction characteristics.

## Conclusions

These results show that the genetic modification of soybean, maize, and cotton by the introduction of genes that confer insect resistance and/or herbicide tolerance does not affect the pollen viability and diameter of these crops studied in Brazil. These results corroborate the current weight of evidence from the previously published literature which support the biosafety profile of GM crops from an ERA perspective. The pollen characterization data contribute to the detailed phenotypic description of GM crops and support the conclusion that the studied events were not fundamentally different from the conventional control and do not pose relevant pathways to impact traits related to reproduction characteristics.

## Supporting information

S1 Table Single and stacked events, conventional control and commercial references planted across different growing seasons and locations for pollen collection. (PDF)
