# Supplementary material for: Genetically modified crops do not present variations in pollen viability and morphology when compared to their conventional counterparts
Source: PLoS One. 2023 May 1;18(5):e0285079. doi: 10.1371/journal.pone.0285079 (PMC10150986; doi:10.1371/journal.pone.0285079)
Supplement: S1 Table — (PDF) [file pone.0285079.s001.pdf]

Crop	Entry description	Material name	Plant cultivation season for pollen collection	Pollen collection and analysis location[1]
Soybean	1. Test	MON 87751	2013	Rolândia, PR
	2. Conventional control	Conventional control		
	3. Commercial reference 1	CD215		
	4. Commercial reference 2	CD202		
	5. Commercial reference 3	NK3363		
	6. Commercial reference 4	Bmx Ativa RR		
Soybean	1. Test	MON 87708	2013	Rolândia, PR
	2. Test	MON 89788		
	3. Test	MON 87708 × MON 89788		
	4. Conventional control	Conventional control		
	5. Commercial reference 1	CD215		
	6. Commercial reference 2	CD202		
	7. Commercial reference 3	NK3363		
	8. Commercial reference 4	Bmx Ativa RR		
Soybean	1. Test	MON 87751 × MON 87701 × MON 87708 × MON 89788 (Soybean stack)	2014	Santa Cruz das Palmeiras, SP
	2. Test	MON 87751		
	3. Test	MON 87701		
	4. Test	MON 89788		
	5. Test	MON 87708		
	6. Conventional control	Conventional control		
	7. Commercial reference 1	CD 202		
	8. Commercial reference 2	NK3363		
	9. Commercial reference 3	Bmx Ativa RR		
	10. Commercial reference 4	Intacta RR2 PRO		
Maize	1. Test	MON 87411	2013	Santa Cruz das Palmeiras, SP
	2. Conventional control	Conventional control		
	3. Commercial reference 1	AG 8025		
	4. Commercial reference 2	AG 9010		
	5. Commercial reference 3	AS 1551		
	6. Commercial reference 4	DKB 390		
Maize	1. Test	MON 87427	2014	Santa Cruz das Palmeiras, SP
	2. Conventional control	Conventional control		
	3. Commercial reference 1	CD 308		
	4. Commercial reference 2	AG 9010		
	5. Commercial reference 3	AS 1551		
	6. Commercial reference 4	DKB 390		
Maize	1. Test	MON 89034 × MIR162	2015	Santa Cruz das Palmeiras, SP
	2. Test	MON 89034		
	3. Conventional control	Conventional control		
	4. Commercial reference 1	CD 308		
	5. Commercial reference 2	AG 9010		
	6. Commercial reference 3	AS 1551		
	7. Commercial reference 4	DKB 390		
Maize	1. Test	MON 87427 × MON 89034 × MIR162 × MON 87411 (Maize stack)	2016	Santa Cruz das Palmeiras, SP
	2. Test	MON 87427		
	2. Test	MON 89034		

	2. Test	MON 87411		
	3. Conventional control	Conventional control		
	4. Commercial reference 1	BALU 184		
	5. Commercial reference 2	DKB 290		
	6. Commercial reference 3	AG 8025		
	7. Commercial reference 4	AS 1551		
Maize	1. Test	MON 87429	2018	Santa Cruz das Palmeiras, SP
	2. Conventional control	Conventional control		
	3. Commercial reference 1	BALU 761		
	4. Commercial reference 2	AG 7088		
	5. Commercial reference 3	AG 8088 PRO2		
	6. Commercial reference 4	DKB 230 PRO3		
Maize	1. Test	MON 95379	2018	Santa Cruz das Palmeiras, SP
	2. Conventional control	Conventional control		
	3. Commercial reference 1	BALU 761		
	4. Commercial reference 2	AG 7088		
	5. Commercial reference 3	AG 8088 PRO2		
	6. Commercial reference 4	DKB 230 PRO3		
Cotton	1. Test	MON 88701	2013	Santa Cruz das Palmeiras, SP
	2. Conventional control	Conventional control		
	3. Commercial reference 1	DP 1228 B2RF		
	4. Commercial reference 2	Nuopal RR		
	5. Commercial reference 3	Delta Opal		
	6. Commercial reference 4	FM 993		
Cotton	1. Test	COT102 × MON 15985 × MON 88913 × MON 88701 (Cotton stack)	2016	Santa Cruz das Palmeiras, SP
	2. Test	MON 88913 × MON 88701		
	3. Conventional control	Conventional control		
	4. Commercial reference 1	Delta Opal		
	4. Commercial reference 2	DP 1227 RF		
	6. Commercial reference 3	TMG 82 WS		

[1]Research station where pollen was collected and analyzed for viability and diameter.
